# Proteomic analysis of the developing mammalian brain links PCDH19 to the Wnt/β-catenin signalling pathway

**DOI:** 10.1038/s41380-024-02482-z

**Published:** 2024-03-07

**Authors:** Rebekah de Nys, Alison Gardner, Clare van Eyk, Stefka Mincheva-Tasheva, Paul Thomas, Rudrarup Bhattacharjee, Lachlan Jolly, Isabel Martinez-Garay, Ian W. J. Fox, Karthik Shantharam Kamath, Raman Kumar, Jozef Gecz

**Affiliations:** 1 Adelaide Medical School, The University of Adelaide, Adelaide, SA Australia; 2 Robinson Research Institute, The University of Adelaide, Adelaide, SA Australia; 3 Genome Editing Program, Adelaide, SA Australia; 4 South Australian Health and Medical Research Institute, Adelaide, SA Australia; 5 Division of Neuroscience, School of Biosciences, Cardiff University, Cardiff, Wales United Kingdom; 6 Australian Proteome Analysis Facility, Macquarie University, Macquarie Park, NSW Australia; 7 School of Biological Sciences, The University of Adelaide, Adelaide, SA Australia

**Keywords:** Neuroscience, Molecular biology, Autism spectrum disorders

## Abstract

Clustering Epilepsy (CE) is a neurological disorder caused by pathogenic variants of the *Protocadherin 19 (PCDH19)* gene. *PCDH19* encodes a protein involved in cell adhesion and Estrogen Receptor α mediated-gene regulation. To gain further insights into the molecular role of PCDH19 in the brain, we investigated the PCDH19 interactome in the developing mouse hippocampus and cortex. Combined with a meta-analysis of all reported PCDH19 interacting proteins, our results show that PCDH19 interacts with proteins involved in actin, microtubule, and gene regulation. We report CAPZA1, αN-catenin and, importantly, β-catenin as novel PCDH19 interacting proteins. Furthermore, we show that PCDH19 is a regulator of β-catenin transcriptional activity, and that this pathway is disrupted in CE individuals. Overall, our results support the involvement of PCDH19 in the cytoskeletal network and point to signalling pathways where PCDH19 plays critical roles.

## Introduction

Clustering Epilepsy (CE), previously known as Girls Clustering Epilepsy (GCE), Female-Limited Epilepsy (FE) or Epilepsy and Mental Retardation Limited to Females (EFMR: OMIM #300088), is a debilitating, early-onset seizure disorder with possible neurological comorbidities [1, 2]. Although this disorder was first described in 1971 [3], the gene responsible for CE, *Protocadherin 19* (*PCDH19*), was eventually identified in 2008 [4]. *PCDH19* consists of 6 exons that encode for a signal peptide, 6 extracellular (EC) cadherin repeats, a transmembrane domain (TM), two conserved motifs in the cytoplasmic domain (CM1 and CM2) and at least one nuclear localisation signal (NLS) (Supplementary Fig. 1) [5–7]. There are two major isoforms of PCDH19 resulting from the alternative splicing of exon 2 (PCDH19 + Ex2 and PCDH19-Ex2) [1]. PCDH19 belongs to the δ2 subclass of the non-clustered protocadherin family that also includes the protocadherins (PCDHs) 8, 10, 17 and 18 [8]. PCDH12 is structurally similar to the other δ2 protocadherins, albeit it lacks the CM1 and CM2 domains [8].

The currently known PCDH19 interacting proteins have stimulated research into the function of this molecule and its potential role in CE. For example, the identification of the interaction between the PCDH19 cytoplasmic region and γ-aminobutyric acid type A receptors (GABA_A_Rs) led to research into the role of PCDH19 in the regulation of GABA_A_R alpha 1 and 2 subunit surface levels and subsequent seizure susceptibility [9]. Our discovery that PCDH19 interacts with the nuclear protein Non-POU Domain Containing Octamer Binding (NONO)/p54nrb provided initial evidence for the role of PCDH19 in Estrogen Receptor (ER) α/NONO-mediated gene regulation [7]. PCDH19 also interacts with LSD1 to regulate the expression of immediate-early genes, further indicating that PCDH19 has a role in the nucleus [6]. Finally, PCDH19 has been found to interact with PCDH10 and PCDH17, N-cadherin (Ncad) and the WAVE regulatory complex (WRC), actin associated proteins and regulators of Rho GTPases to influence cell adhesion, mitosis and actin cytoskeleton dynamics [8, 10–14]. Overall, research into the PCDH19 interactome will pave the way for better understanding of the molecular role of PCDH19 in CE pathology.

More recent proteomic studies have found that PCDH19 plays a role in Wnt/β-catenin signalling. A proximity-dependent biotinylation study in HEK293T cells expressing BioID-fused zebrafish Pcdh19 identified several β-catenin binding proteins as interactors of Pcdh19 [11]. Another study showed that zebrafish Pcdh1a, Pcdh9, Pcdh18b and Pcdh19 can interact with the non-canonical Wnt receptor Receptor-like Tyrosine Kinase (Ryk) and that *pcdh19−/−* zebrafish have increased expression of β-catenin target genes *Lef1* and *Axin* when compared to *pcdh19*+*/+* zebrafish [15].

Whereas identification of PCDH19 interacting proteins has increased our understanding of the molecular function of PCDH19, the PCDH19 interactome has not been studied in the developing mammalian brain, a disease-relevant organ. To address this gap, we performed Liquid Chromatography-Tandem Mass Spectrometry (LC-MS/MS) based analysis on PCDH19 and its interacting proteins, purified from the hippocampi and cortices of embryonic and postnatal Pcdh19-HA-FLAG mice. Our results showed an enrichment of proteins involved in reported PCDH19-associated processes (cytoskeleton organisation and actin filament-based process) [11] as well as likely novel pathways. We report the novel and validated interacting proteins Capping Actin Protein of Muscle Z-Line Subunit Alpha 1 (CAPZA1), αN-catenin (CTNNA2) and β-catenin (CTNNB1). Importantly, we show that PCDH19 is a regulator of β-catenin transcriptional activity, with dysregulation of this pathway also observed in CE patient skin cells. These results provide valuable insights into the molecular function of PCDH19 and its role in CE.

## Methods

### Cell culture

HEK293T cells (ATCC, 293T-CRL-3216) were maintained in DMEM with GlutaMAX (Gibco) and 10% Heat Inactivated Foetal Bovine Serum (HI-FBS) (Gibco) and were mycoplasma free. Primary skin fibroblasts were maintained in RPMI-1640, GlutaMAX (Gibco) and 10% HI-FBS. All cells were cultured at 37 °C, 5% CO_2_.

### Mouse experimental model

All mouse work was conducted following approval by South Australian Health and Medical Research Institute (SAHMRI) (SAM312; SAM20-015) and The University of Adelaide Animal Ethics Committee (AEC No. #1200) in accordance with the Australian code for the care and use of animals for scientific purposes. Generation and characterisation of the Pcdh19-HA-FLAG mice was reported previously [12]. Experiment 1: Cortices from two pooled E18.5 male and female Pcdh19-HA-FLAG or two pooled PCDH19 untagged mice were dissected and dounced to homogenise in IP Buffer (50 mM Tris-HCl pH 7.5, 150 mM NaCl, 0.2% Triton-X-100, 2 mM EDTA, 0.01% SDS, 50 mM NaF, 0.1 mM Na_3_VO_4_, 1× Protease Inhibitor/No EDTA). The tissue was sonicated twice (30% Amplitude, Pulse 15 s; Vibra-Cell VCX 130, Sonics) and lysate cleared of cellular debris by centrifugation at 14,000 × *g*, at 4 °C. 5 mg of protein was incubated with 80 μL packed volume of mouse anti-FLAG M2 affinity gel (Sigma-Aldrich, cat #A2220) overnight at 4 °C with rotation. The next day, the beads were washed three times with IP Buffer and three times with 20 mM Tris-HCl pH 7.5 buffer. The protein was eluted in 100 μL 1×SDS buffer (62.5 mM Tris-HCl pH 6.8, 2% SDS, 10% glycerol) [16] at 95 °C/5 minutes. Experiment was performed in duplicate. Experiment 2: Cortices and hippocampi from 16-40 E17.5-18.5 or 7-10 P6-7 male and female Pcdh19-HA-FLAG mice were dissected and dounced to homogenise in IP Buffer. The tissue was sonicated twice (30% Amplitude, Pulse 15 s) and lysate cleared by centrifugation. 5 mg of protein was incubated with 80 μL packed volume of mouse anti-FLAG M2 affinity gel (Sigma-Aldrich, cat #A2220) or 160 μL packed volume of mouse anti-IgG affinity gel (Sigma-Aldrich, cat #A0919) overnight at 4°C with rotation. The washing of the beads and elution of the protein was performed like Experiment 1. PCDH19-HA-FLAG IP was confirmed by western blotting. IPs were performed in 2–4 biological replicates per developmental stage. Mouse sample sizes were chosen to ensure enough cortices-hippocampi protein would be obtained for IP.

### Liquid chromatography-tandem mass spectrometry (LC-MS/MS) analysis

LC-MS/MS was performed by the Australian Proteome Analysis Facility (Macquarie University, NSW). Samples were resolved on 4–20% SDS-PAGE gels at 15 mA for 22 min, gels fixed with 10% methanol 7% acetic acid, stained with Coomassie stain, the bands excised, in-gel trypsinised overnight, peptides extracted and analysed by LC-MS/MS. The raw data files were converted to Mascot generic files and searched against mouse SwissProt database with <1% false discovery rate. *P* values for non-redundantly identified proteins were calculated with paired t-test and fold-change was calculated by log transformation and subtraction from the control (IgG or untagged mouse).

### Western blotting

Cell lysates were denatured and resolved by 6% homemade SDS-PAGE [16]. Proteins were transferred onto nitrocellulose membranes, blocked with 10% skim milk/1× TBST and probed with mouse anti-FLAG (Sigma-Aldrich, cat #F3165), mouse anti-HA (Sigma-Aldrich, cat #H3663), mouse anti-V5 (Thermo Fisher, cat #46-0705), mouse anti-Myc (Sigma-Aldrich, cat #M4439), mouse anti-β-catenin (BD Transduction Laboratories, cat #610154), rabbit anti-PCDH19 (Bethyl, cat #A304-468A), rabbit anti-β-Tubulin (Abcam, cat #ab6046), or mouse anti-N-cadherin (Transduction Laboratories, cat #C70320) in 2% skim milk/1× TBST overnight at 4 °C. The next day, the membranes were washed and probed with goat anti-mouse immunoglobulins/HRP (Dako, cat #P0447) or goat anti-rabbit immunoglobulin/HRP (Dako, cat #P0448) with 2% skim milk/1× TBST, washed with 1× TBST and detected by Clarity Western enhanced chemiluminescence (ECL) Substrate (Bio-Rad) using BioRad ChemiDoc MP Imaging System with Imaging Lab 5.0.

Western Blotting in Supplementary Fig. 8C was performed by resolving denatured cell lysates by NuPAGE gels (Invitrogen). Proteins were transferred onto nitrocellulose membranes, blocked with EveryBlot Blocking buffer (BioRad) and probed with rat anti-HA (Roche, cat # 12158167001) or chicken anti-GAPDH (Abcam, cat #Ab15822) in EveryBlot Blocking buffer overnight at 4 °C. The next day, the membranes were washed and probed with goat anti-rat IgG HRP conjugate (R&D Systems, cat # HAF005)) or goat anti-chicken IgG HRP conjugate (Abcam, cat # Ab6877) in EveryBlot Blocking buffer, washed with 1× TBST and detected by WesternBright Sirius Chemiluminescent Detection Kit (Advansta) using BioRad ChemiDoc XR+ Imaging System with ImageLab software 6.0.1.

### Generation of pCMV-HA-CTNNA2,pCMV-HA-CAPZA1 and pCMV-HA-CDH2 expression constructs

*CTNNA2*,* CAPZA1* and *CDH2* were PCR amplified from human brain cDNA by SuperFi PCR Master Mix (Thermo Fisher) using the following conditions: one cycle 98 °C 30 s; 40 cycles of 98 °C 15 s, 59 °C 15 s, 72 °C 60 s; 72 °C 10 min. *CTNNA2*,* CAPZA1* and *CDH2* open reading frames were cloned into pCMV-HA at *Hin*dIII+*Not*I*, Eco*RV*+Hin*dIII and *Eco*RV+*Xho*I restriction sites, respectively. See Supplementary Methods Table for primer sequences. pCMV-Myc-PCDH19, pCMV-Myc-PCDH19-FLAG, pCMV-PCDH10-Myc/HA, pCMV-PCDH12-Myc/HA and pCMV-PCDH17-Myc expression constructs were reported previously [7, 12].

### Co-immunoprecipitation of epitope tagged proteins

HEK293T cells were plated at 5 × 10^5^ cells/well in 6-well culture dishes. The next day the cells were transfected with the appropriate tagged expression constructs using Lipofectamine 3000 reagent. The cells were harvested 24 h later and lysed in IP buffer by sonication (Sonic’s Vibra-Cell VCX). The lysate was clarified by centrifuging at 15,800 × *g*, 20 min, 4 °C and the supernatants incubated with anti-c-Myc-Magnetic Dynabeads (Thermo Fisher, cat #88843), anti-HA agarose beads (Sigma Aldrich, cat #E6779) or anti-FLAG M2 agarose beads (Sigma Aldrich, cat #A2220) overnight at 4 °C. Next day, the beads were washed three times with IP Buffer and twice with 20 mM Tris-HCl pH 7.5. The proteins were eluted in 40 µL 1× SDS protein-loading buffer (62.5 mM Tris-HCl, pH 6.8, 2% SDS, 10% glycerol, 5% β-mercaptoethanol) at 95 °C for 5 min [16]. IPs were performed in two biological replicates.

### pCMV-PCDH17-Myc co-immunoprecipitation

Cell plating, transfections and co-IP were carried out as mentioned above (*pCMV-HA-αN-catenin and pCMV-HA-CAPZA1 co-immunoprecipitation*) in IP buffer previously described [12].

### β-catenin co-immunoprecipitation

For PCDH19 immunoprecipitation from postnatal (P5-6) Pcdh19-HA-FLAG mouse brain, combined cortical and hippocampal tissue was dissected and homogenised in IP Buffer (50 mM Tris-HCl pH 7.5, 150 mM NaCl, 0.2% Triton-X-100, 2 mM EDTA, 2× Protease Inhibitor/no EDTA). The tissue was sonicated twice (30% Amp for 15 s) and lysate clarified by centrifuging at 21,100 × *g*, 20 min at 4 °C. Equal amounts of protein were incubated with 80 μL packed volume of mouse anti-FLAG M2 (Sigma-Aldrich, cat #A2220) or mouse anti-IgG affinity gel (Sigma-Aldrich, cat #A0919) overnight at 4 °C with rotation. The next day, the beads were washed four times with Wash Buffer (50 mM Tris-HCl pH 7.5, 150 mM NaCl, 0.2% Triton-X-100, 2 mM EDTA, 2× Protease Inhibitor/no EDTA, 0.1% SDS) and then twice with 20 mM Tris-HCl pH 7.5 buffer. The protein was eluted in 100 μL 1× SDS loading buffer (62.5 mM Tris-HCl, pH 6.8, 2% SDS, 10% glycerol, 5% β-mercaptoethanol) [16] at 95 °C for 5 min and stored at −80 °C until western blot analysis. Western blots were probed with rabbit anti-PCDH19 (Bethyl, cat #A304-468A) and mouse anti-β-catenin (BD Transduction Laboratories, cat #610154) antibodies. IPs were performed in four biological replicates.

### Myc-PCDH19-FLAG/β-catenin-V5 and Myc-PCDH19-FLAG/β-catenin-V5/CDH2-HA co-immunoprecipitation

4 × 10^5^ HEK293T cells were plated in six well culture dishes in DMEM, 10% FBS and incubated at 37 °C/5% CO_2_ overnight. The cells were transfected with the appropriate tagged expression plasmids using Lipofectamine 3000 transfection reagent and incubated overnight. Next day the cells were harvested and lysed by sonication (20% Amp for 12 s) in IP Buffer. The lysate was clarified by centrifuging at 21,100 × *g*, 20 min, 4 °C and incubated with 25 µL packed volume anti-FLAG agarose beads (Sigma-Aldrich) for 1 hour at 4 °C. The beads were washed four times with Wash Buffer and then twice with 20 mM Tris-HCl pH 7.5 buffer and eluted in 40 µL 1×SDS loading buffer at 95 °C for 5 min and stored at −80 °C until western blot analysis. Western blots were probed with mouse anti-Myc (Sigma-Aldrich, cat #M4439), mouse anti-HA (Sigma-Aldrich, cat #H3663) and mouse anti-V5 (Thermo Fisher, cat #46-0705) antibodies. IPs were performed in three biological replicates.

### Immunofluorescence microscopy

Cryosections of dissected P6 *Pcdh19*^*HA-FLAG/HA-FLAG*^ mouse brains were prepared as previously described [17]. Cryosections were permeabilized with 0.5% Triton-X-100/1× PBS and washed with Wash Solution (0.05% Triton-X-100, 1× PBS). The cryosections were blocked with Blocking Buffer (0.1% Triton-X-100, 1% BSA, 10% Horse Serum, 1× PBS) for 1 h at room temperature and probed with rabbit anti-HA (1:400, Cell Signalling, cat #14793S) and mouse anti-β-catenin (1:500, BD Transduction Laboratories, cat #610154) in Blocking Buffer overnight at 4 °C. The next day, the cryosections were washed three times with Wash Buffer and incubated with donkey alexa-448 conjugated anti-rabbit (Invitrogen, cat #A21206) and donkey alexa-555 conjugated anti-mouse (Invitrogen, cat #A31570) (1:1500) in Blocking Buffer. The cryosections were washed three times with Wash Buffer and sealed with coverslips using ProLong Diamond Antifade Mountant with DAPI (Thermo Fisher). Slides were viewed at 20× magnification on Olympus IX71 inverted fluorescence microscope.

Background fluorescence signal in each image was corrected using Fiji’s Process background function (NIH, USA) and then Costes automatic thresholding [18] was applied using the JACoP plugin for Fiji (https://imagej.nih.gov/ij/plugins/track/jacop2.html). PCDH19-HA (green channel) and β-catenin (red channel) colocalization was quantified using the JACoP plugin across the whole image area and cytofluorograms representing colocalization spread were generated. Pearson’s correlation coefficient (r) was calculated, where ‘r’ indicates the estimate of the goodness of fit of the linear regression model and reflects the quantitative measure of colocalization of two fluorochromes. ‘r’ ranges between 1 to −1, with 1 representing a positive correlation and −1 representing a negative correlation. r = 0 represents absence of correlation [19] For each brain region, at-least 3 images were analysed to calculate the Pearson’s correlation for colocalization of PCDH19-HA and β-catenin.

### TOP-Flash luciferase reporter assay

8 × 10^4^ HEK293T cells/well were plated into a 24 well culture dish. Next day, the cells were transfected with 10 ng pRL-TK, 200 ng TOP or FOP reporter plasmid and 500 ng pCMV empty vector or pCMV-Myc-PCDH19 plasmids [500 ng WT+Ex2, WT-Ex2, p.N340S (c.1019A>G), V441E (c.1322T>A), p.D375N (c.1123G>A), EC, EC-TM or 150 ng CD] using Lipofectamine 3000 transfection reagent. Three hours later the media was changed to DMEM with 0.5% FBS and treated with 100 ng/mL human Wnt3a ligand or vehicle (0.1% BSA/1× PBS) for 18 hours. Luciferase assay was performed using Dual-Luciferase Reporter Assay System (Promega) and the GloMax 20/20 Luminometer. Firefly luciferase values were normalised to *Renilla* luciferase values and expressed as Relative Light Units (RLU). The luciferase activity was normalised to FOP control. Luciferase reporter assays were performed in three technical and three biological replicates. The luciferase activity was normalised to FOP control. Graphs display the mean and standard deviation (SD). Statistical analysis performed using One-Way ANOVA with Sidak correction for multiple comparisons and 95% confidence interval.

To investigate the role of mouse PCDH19, 5 × 10^4^ HEK293T cells/well were plated in a 24-well plate. Next day, cells were transfected with 150 ng M50 Super 8× TOP-Flash plasmid (Addgene), 5 ng pRL *Renilla* vector (Promega) and the appropriate mouse pCBA-PCDH19-HA plasmids (150 ng pCBA, PCDH19 WT+Ex2, CD, CD-ΔCM1, CD-ΔCM2 or CD-NLS K779-780A mutant)), using Lipofectamine 3000 transfection reagent. Cells were treated with 200 ng human Wnt3a ligand or vehicle (1× PBS) for 24 h. Firefly and *Renilla* luminescence activities were assayed using the Dual-Luciferase Reporter Assay System (Promega). Measurements were performed with a FLUOstar Omega microplate reader (BMG Labtech). Firefly luciferase values were normalised to *Renilla* luciferase values and expressed as RLU. Luciferase reporter assays were performed in two technical and seven biological replicates. Graph displays the mean and standard deviation (SD). Statistical analysis performed using Two-Way ANOVA and 95% confidence interval.

### RNA-sequencing

RNA sequencing was performed by Azenta Biotech. Poly A+ enriched patient fibroblast total RNA samples were used for preparing cDNA libraries and subjected to strand-specific sequencing at 40-60 million 2 × 150 paired-end reads per sample. Transcript-level abundances were quantified using Salmon against the pre-built Refgenie hg38 indices (http://refgenomes.databio.org/v3/assets/splash/2230c535660fb4774114bfa966a62f823fdb6d21acf138d4/salmon_sa_index), and aggregated to gene level counts using the tximport function of DESeq2 in R v4.1.2. Differential gene expression analysis was performed using DESeq2 v1.34.0 [20].

### RNA extraction and Reverse Transcription-quantitative PCR (RT-qPCR)

RNA was extracted using QIAshredder and RNeasy Kit (Qiagen) and on-column RNase-free DNase treatment according to manufacturer’s protocol. 2 µg RNA was reverse transcribed using SuperScript IV (Thermo Fisher). qPCRs for *HPRT1* (Life Technologies, 4325801) and *FZD3* (ThermoFisher, Hs00907280_m1) were performed using TaqMan probes with Taqman Gene Expression Master Mix (2×) (Thermo Fisher) and diluted cDNA using the following cycling conditions: hold at 50 °C for 2 min and at 95 °C for 10 min followed by 40 cycles of 95 °C for 15 s and 60 °C for 1 min. Data was acquired using StepOne Real-Time PCR System and software V 2.0 (Applied Biosystems) and expression of the target gene was normalised against *HPRT1*. Graph displays the mean and standard deviation (SD). Statistical analysis was performed using unpaired t-test with Welch’s correction and 95% confidence interval.

### Enrichment analysis

Gene ontology enrichment analysis, molecular function, cellular component, and biological process - was performed using ShinyGO 0.76.3 (http://bioinformatics.sdstate.edu/go/) with FDR cutoff 0.05, pathway size Min. 2 and Max. 2000 [21]. Transcription factor target enrichment analysis of AF vs. FC dysregulated genes was performed using ShinyGO 0.76.3 with TF.Target.TFact database. Predicted subcellular component localisation was curated from UniProt (https://www.uniprot.org/) [22]. Signalling pathway enrichment analysis was performed using Enrichr (https://maayanlab.cloud/Enrichr/) with the Panther metabolic and signalling pathway database [23].

## Results

### The PCDH19 interactome

#### PCDH19 interactome is enriched for actin and cytoskeleton binding proteins in the mammalian brain

**Fig. 1:**
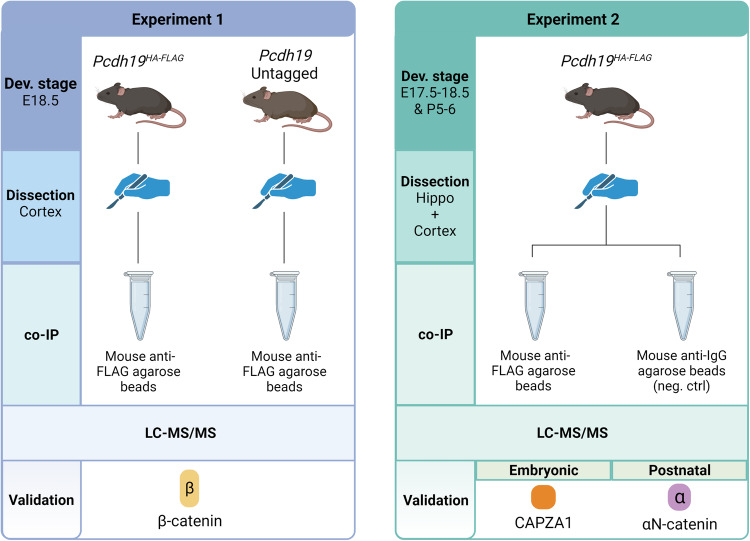
Schematic diagram of the experimental plan. Experiments differed based on tissue, developmental stage and controls used. Experiment 1: Cortices from E18.5 *Pcdh19*^*HA-FLAG*^ and *Pcdh19* untagged negative control mice. Experiment 2: Combined hippocampi and cortices from E17.5-18.5 and P5-6 *Pcdh19*^*HA-FLAG*^ mice. PCDH19-HA-FLAG was IPed using mouse anti-FLAG agarose beads in both experiments. Mouse anti-IgG agarose beads were used as a negative control for Experiment 2. β-catenin (identified in experiment 1), αN-catenin and CAPZA1 (identified in experiment 2) were validated by co-IPs in transfected HEK293T cells (see Fig. 3).

To investigate the PCDH19 interactome and identify novel and disease-relevant interacting proteins, we performed two independent Liquid Chromatography-Tandem Mass Spectrometry (LC-MS/MS) experiments of the developing mouse brain (Fig. 1).

For the first experiment, we performed LC-MS/MS of PCDH19 immunoprecipitated (IPed) from the cortices of the HA-FLAG-tagged or untagged (control) embryonic (E18.5) mice. After strict filtering (interacting proteins in two biological replicates with a t-test *p* value $\le$ 0.05 and abundance difference Fold Change $\ge$ 1.5), we identified six potential PCDH19 interacting proteins (ATP1A1, ATP1B1, MAP1A, SHANK2, SLC25A4 and SORBS2) (Table 1, Supplementary Table 1).

**Table 1:** Identification of novel PCDH19 interacting proteins in the mouse brain.

Experiment 1		Experiment 2			
Embryonic		Embryonic		Postnatal	
Gene ID	Protein name	Gene ID	Protein name	Gene ID	Protein name
ATP1A1	ATPase Na + /K+ transporting subunit alpha 1	ANK2	Ankyrin 2	ABLIM1	Actin Binding LIM Protein 1
ATP1B1	ATPase Na + /K+ transporting subunit beta 1	CAPZA1^b^	Capping Actin Protein Of Muscle Z-Line Subunit Alpha 1	ACTN1	Actinin Alpha 1
MAP1A	Microtubule-associated protein 1A	CAPZA2	Capping Actin Protein Of Muscle Z-Line Subunit Alpha 2	ATP5F1A	ATP synthase F1 subunit alpha
SHANK2	SH3 And Multiple Ankyrin Repeat Domains 2	CAPZB	Capping Actin Protein Of Muscle Z-Line Subunit Beta	CAPZA2	Capping Actin Protein Of Muscle Z-Line Subunit Alpha 2
SLC25A4	Solute carrier family 25 member 4	CEP170	Centrosomal Protein 170	CLASP2^a^	Cytoplasmic Linker Associated Protein 2
SORBS2	Sorbin and SH3 domain containing 2	CLASP2^a^	Cytoplasmic Linker Associated Protein 2	CRMP1	Collapsin Response Mediator Protein 1
		DBN1	Drebrin 1	CTNNA2^b^	Catenin Alpha 2
		EIF4B	Eukaryotic translation initiation factor 4B	CTNND2	Catenin Delta 2
		FLNA	Filamin A	DPYSL3	Dihydropyrimidinase Like 3
		GAPDH	Glyceraldehyde-3-Phosphate Dehydrogenase	EIF4B	Eukaryotic Translation Initiation Factor 4B
		KCTD5	Potassium Channel Tetramerization Domain Containing 5	FUS	FUS RNA Binding Protein
		LIMA1	LIM Domain And Actin Binding 1	GAPDH	Glyceraldehyde-3-Phosphate Dehydrogenase
		MAP1B	Microtubule Associated Protein 1B	HDAC6	Histone deacetylase 6
		MAP2	Microtubule Associated Protein 2	JAK1	Janus Kinase 1
		MYO18A	Myosin XVIIIA	KCTD5	Potassium Channel Tetramerization Domain Containing 5
		PPM1B	Protein phosphatase 1B	MAP1B	Microtubule Associated Protein 1B
		PPP1R12B	Protein Phosphatase 1 Regulatory Subunit 12B	MAP2	Microtubule Associated Protein 2
		PPP1R9B	Protein Phosphatase 1 Regulatory Subunit 9B	MYH14	Myosin Heavy Chain 14
		PRMT5	Protein arginine methyltransferase 5	MYO18A	Myosin XVIIIA
		PRPF31	Pre-mRNA Processing Factor 31	PPM1B	Protein phosphatase 1B
		PSMD2	26S proteasome non-ATPase regulatory subunit 2	PPP1R9A	Protein Phosphatase 1 Regulatory Subunit 9A
		RPL29	Ribosomal Protein L29	PRMT5	Protein arginine methyltransferase 5
		RPL8	Ribosomal Protein L8	RPL8	Ribosomal Protein L8
		SCYL2	SCY1 Like Protein 2	SIPA1	Signal-Induced Proliferation-Associated 1
		SOGA3	SOGA Family Member 3	SOGA3	SOGA Family Member 3
		SPTAN1^a^	Alpha II-spectrin	SPTAN1^a^	Alpha II-spectrin
		TRIOBP	TRIO and F-actin Binding Protein	SPTBN1	Beta II-spectrin
		VIM	Vimentin	SRRM2	Serine/Arginine Repetitive Matrix 2
				THRAP3	Thyroid Hormone Receptor Associated Protein 3
				ZC2HC1A	Zinc Finger C2HC-Type Containing 1A

List of PCDH19 interacting proteins identified by LC-MS/MS in the mouse brain (t-test *p* ≤ 0.05 and FC ≥ 1.5).

^a^Previously published interactor.

^b^Interactor validated in our study.

**Fig. 2:**
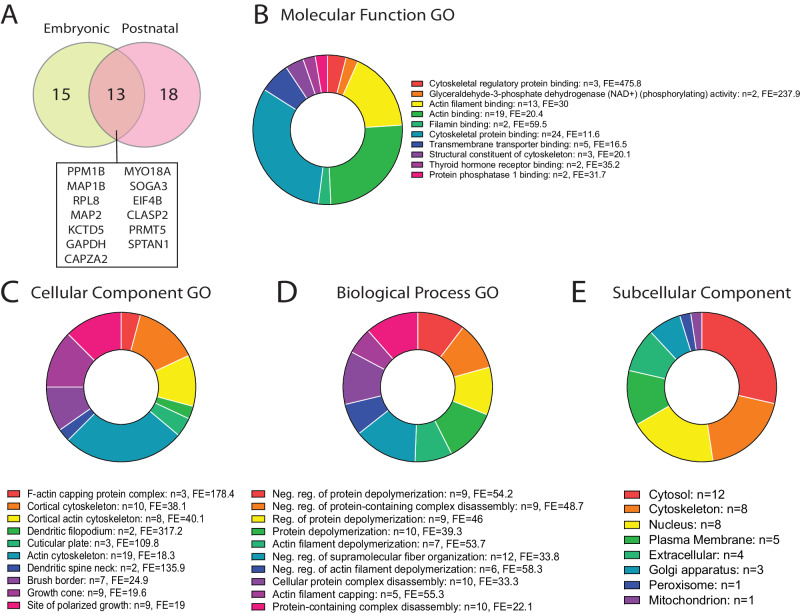
The PCDH19 interactome in the developing mouse brain. **A** Venn diagram showing the overlap of candidate interacting PCDH19 proteins identified in the embryonic and postnatal brain in Experiment 2. **B** Molecular function, **C** Cellular component, **D** Biological Process [21], and **E** predicted subcellular component analysis [22] of PCDH19 interacting proteins identified in the mouse brain (Experiments 1 and 2). The graphs show the top 10 enriched gene ontology (GO) pathways sorted by FDR and Fold enrichment. n number of genes, FE fold enrichment.

In the second experiment, we aimed to investigate the PCDH19 interactome across developmental stages and using an alternative approach. Here, the PCDH19-HA-FLAG proteins were IPed from the total protein lysate of embryonic (E17.5-18.5) or postnatal (P6-7) hippocampi and cortices using mouse anti-FLAG and control IgG agarose beads. These stages of development are ideal for interactor studies due to high expression of PCDH19 in the cortex [24]. Detection of PCDH19 and its known interactor N-cadherin confirmed that our IP conditions were optimal for successfully immunoprecipitating PCDH19 and interacting proteins (Supplementary Fig. 2). LC-MS/MS of these IPed products identified 27 embryonic and 32 postnatal PCDH19 interacting proteins in at least two technical replicates and one biological replicate with a t-test p value $\le$ 0.05 and Fold Change $\ge$ 1.5 when compared to IgG control (Table 1 and Supplementary Table 2). 13 of these proteins were identified both in embryonic and postnatal stages, for example the microtubule binding protein CLASP2 (Fig. 2A). This protein was previously shown to interact with PCDH19 by proximity-dependent biotinylation assay in HEK293T cells transfected with zebrafish Pcdh19 [11]. The protein SPTAN1 was also identified as a PCDH19 interacting protein in both developmental stages. Previous crosslinking-MS experiments in U2OS cells showed SPTAN1 and PCDH19 interaction [25]. In addition, our results show that PCDH19-CLASP2 and PCDH19-SPTAN1 also interact in the mouse hippocampus/cortex at the two development stages (Table 1).

We performed pathway enrichment analysis to determine if our identified proteins were specifically associated with certain biological processes (Fig. 2B–E). Similar to previous studies, our results showed enrichment for proteins involved in actin binding and cytoskeleton processes [11] (Fig. 2B). Many identified interacting proteins also have roles in regulation of protein depolymerisation and are located in the actin cytoskeleton (Fig. 2C, D). The majority of the PCDH19 interacting proteins were found to localise to the cytoplasm, cytoskeleton, cell membrane and nucleus, which is consistent with the known localisation and function of PCDH19 (Fig. 2E) [6, 7, 11].

We searched the OMIM database (https://www.omim.org/) to determine if any of the potential PCDH19 interactors are associated with neurological disorders. 21 of these proteins were associated with a disorder recorded in OMIM, including one (*SPTAN1*) implicated in Developmental and Epileptic Encephalopathy 5 (DEE5) that has overlapping clinical features, such as early onset seizures, ID and developmental delay, with CE (Supplementary Table 3).

#### Meta-analysis of known PCDH19 interacting proteins to map the PCDH19 interactome

To date, researchers have employed various methodologies and models to identify novel PCDH19 interacting proteins [7, 10, 11, 13]. To gain a comprehensive overview of the PCDH19 interactome, we performed a meta-analysis of all published PCDH19 interacting proteins. We identified 13 studies that detected a PCDH19 interacting protein. Together with our LC-MS/MS results, we postulate that there are 252 unique PCDH19 interacting proteins reported in the literature and this study (Supplementary Table 4). The majority of these proteins were identified using human cell lines rather than brain tissue and through the use of large-scale proteomic studies with few experimentally validated interactors (Supplementary Fig. 3A). All proteins identified in the mouse brain come from this study, with an additional 2 proteins identified in chicken brain (NAP1 and CYFIP2) and 5 proteins identified in rat brain (HDAC2, RCOR1, SRF, LSD1 and GABRA1) [6, 9, 13]. In addition to SPTAN1 and CLASP2, CEP170 and CYFIP2 were reported in multiple studies [11, 13]. We performed gene ontology enrichment analysis to map the known PCDH19 proteome. This revealed enrichment of many of the same pathways identified by us (i.e., cytoskeletal organisation, actin filament-based processes and neurogenesis) (Supplementary Fig. 3B–E). Enrichment analysis of signalling pathways identified Wnt, cadherin and angiogenesis signalling as the most enriched pathways for PCDH19-associated proteins (Supplementary Fig. 3F).

### PCDH19 is implicated in Wnt/β-catenin signalling

#### PCDH19 interacts with αN-catenin, CAPZA1 and β-catenin

**Fig. 3:**
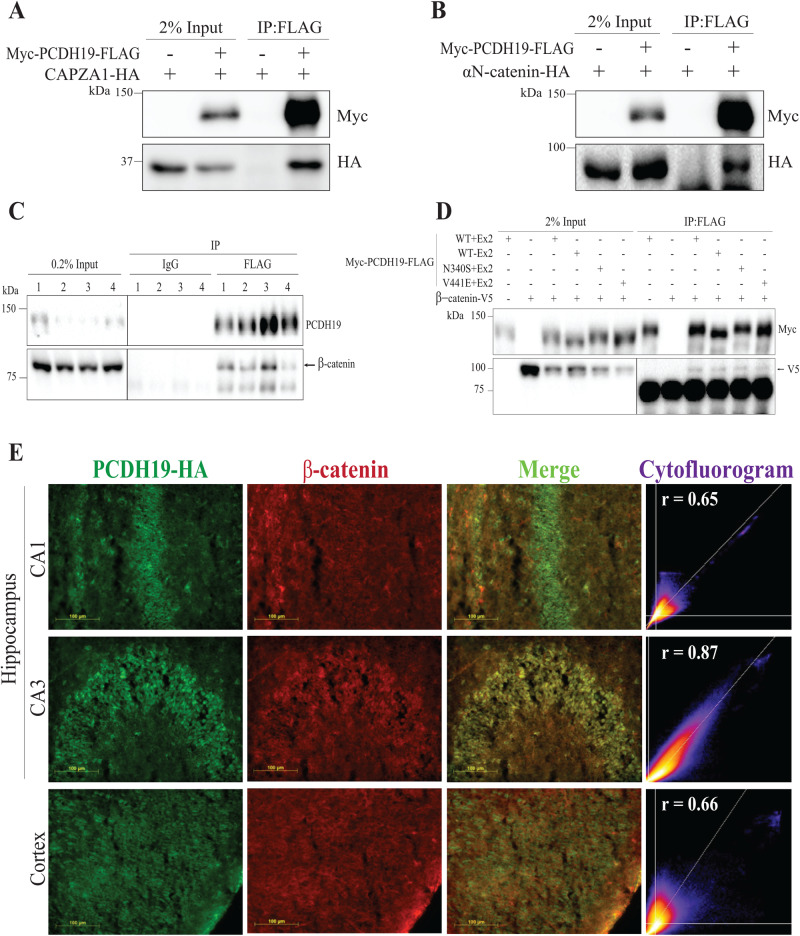
Validation of PCDH19 interacting protein in HEK293T cells. Myc-PCDH19-FLAG was immunoprecipitated with anti-FLAG agarose beads from the HEK293T cells ectopically expressing the epitope-tagged proteins as shown. 2% of inputs and 25% of IP samples were western blotted to detect (**A**, **B** and **D**) Myc-PCDH19-FLAG, (**A**) CAPZA1-HA, (**B**) αN-catenin-HA and (**D**) β-catenin-V5. **C** PCDH19-HA-FLAG was immunoprecipitated with mouse anti-FLAG antibody or mouse IgG agarose beads from P5-6 mouse hippocampi/cortices. 0.2% of inputs and 20% IP samples were western blotted for endogenous PCDH19-HA-FLAG and β-catenin proteins. Experiment was performed in quadruplicate. **E** Representative PCDH19-HA (green) and β-catenin (red) immunostaining of P6 *Pcdh19*^*HA-FLAG/HA-FLAG*^ brain section. Cytofluorograms with Pearson’s correlation co-efficient (r) for colocalization of PCDH19-HA and β-catenin across the whole image area is shown.

Our results as well as previous studies have shown many PCDH19 interacting proteins are involved in Wnt/β-catenin signalling, cell adhesion and cytoskeleton binding [10, 11, 15]. Furthermore, our LC-MS/MS data indicated β-catenin to be a potential PCDH19 interactor, though it was not observed to be statistically significantly altered (Experiment 1, *p* = 0.44, FC = 2.70). Therefore, we validated CAPZA1, αN-catenin and β-catenin interaction with PCDH19 in vitro due to their roles in actin cytoskeleton remodelling, cell adhesion and gene expression regulation, respectively (Fig. 3A, B, D) [26–29]. Considering the role of β-catenin in Wnt/β-catenin signalling and cell adhesion [30], we were particularly interested in the interaction of PCDH19 with β-catenin. Therefore, we performed co-IP assays on the combined hippocampi/cortices of P6 Pcdh19-HA-FLAG mice and confirmed the PCDH19-β-catenin interaction in the mouse brain (Fig. 3C). To determine if this interaction is affected by PCDH19 isoform [1] or CE-variant, we performed co-IP assays in HEK293T cells transfected with epitope tagged β-catenin and either the isoforms PCDH19 with Exon 2 (+Ex2), PCDH19 without Exon 2 (-Ex2), or recurrent CE pathogenic variants p.N340S [2] or p.V441E [4]. PCDH19 missense constructs expressed the protein with Exon 2 (+Ex2). We observed that β-catenin interacts with PCDH19 regardless of isoform or CE-missense variant (Fig. 3D). Immunofluorescence of P6 *Pcdh19*^*HA-FLAG/HA-FLAG*^ mouse brain sections suggests co-expression of PCDH19 and β-catenin in the hippocampus and cortex (Fig. 3E). Overall, our proteomic analysis identified PCDH19 interaction with CAPZA1 and αN-catenin in human cells and, importantly, interaction and co-expression of PCDH19 and β-catenin in mouse brain and human cells.

#### β-catenin interacts with PCDH10 and PCDH17, but not PCDH12

The protocadherin family members can be divided into groups based on genomic location and protein structure [8]. Clustered protocadherins are encoded in tandem at chromosome 5q31, while non-clustered protocadherins are encoded throughout the genome and mainly comprise of δ-protocadherins [31]. Proteins of the δ2-protocadherin subfamily (PCDH8, PCDH10, PCDH17, PCDH18 and PCDH19) have six extracellular cadherin repeats and two conserved cytoplasmic motifs: CM1 and CM2 [8]. PCDH12 is phylogenetically related to the δ2-protocadherin subfamily but lacks CM1 and CM2 [32, 33]. To determine if CAPZA1, αN-catenin and β-catenin interact with other δ2-protocadherins, we performed co-IP assays in HEK293T cells transfected with epitope tagged CAPZA1, αN-catenin or β-catenin and either PCDH10, PCDH12 or PCDH17. We were unable to detect an interaction between CAPZA1 or αN-catenin and the protocadherins tested in our IP buffer conditions (Supplementary Fig. 4A–F). However, we found that β-catenin interacts with PCDH10 and PCDH17, but not PCDH12 (Supplementary Fig. 4G–I). As PCDH19, PCDH10 and PCDH17 share the conserved CM1 and CM2 regions, we hypothesised that PCDH19 interaction with β-catenin occurs through the cytoplasmic domain (CD). To test this, we performed co-IP assays using HEK293T cells expressing epitope-tagged cytoplasmic (CD), extracellular (EC) or extracellular-transmembrane (EC-TM) fragments of PCDH19 protein. Our results showed immunoprecipitation of β-catenin with all the three protein regions (Supplementary Fig. 5). We predict that PCDH19 interacts either directly or indirectly (via another protein) with β-catenin via multiple domains.

As both PCDH19 and β-catenin are known to interact with N-cadherin, it is likely that the PCDH19-β-catenin interaction is mediated via N-cadherin, as has been proposed before [34]. We explored this possibility by co-immunoprecipitating Myc-PCDH19-FLAG full length (FL) or Myc-PCDH19-EC-FLAG and β-catenin-V5 from HEK293T cells with or without N-cadherin-HA expression. Our results showed that N-cadherin interacts with PCDH19-FL and EC and increases their interaction with β-catenin. This suggests that N-cadherin, perhaps in addition to other unknown protein/s, contributes to PCDH19-FL or PCDH19-EC interaction with β-catenin (Supplementary Fig. 6).

#### PCDH19 regulation of β-catenin transcriptional activity

**Fig. 4:**
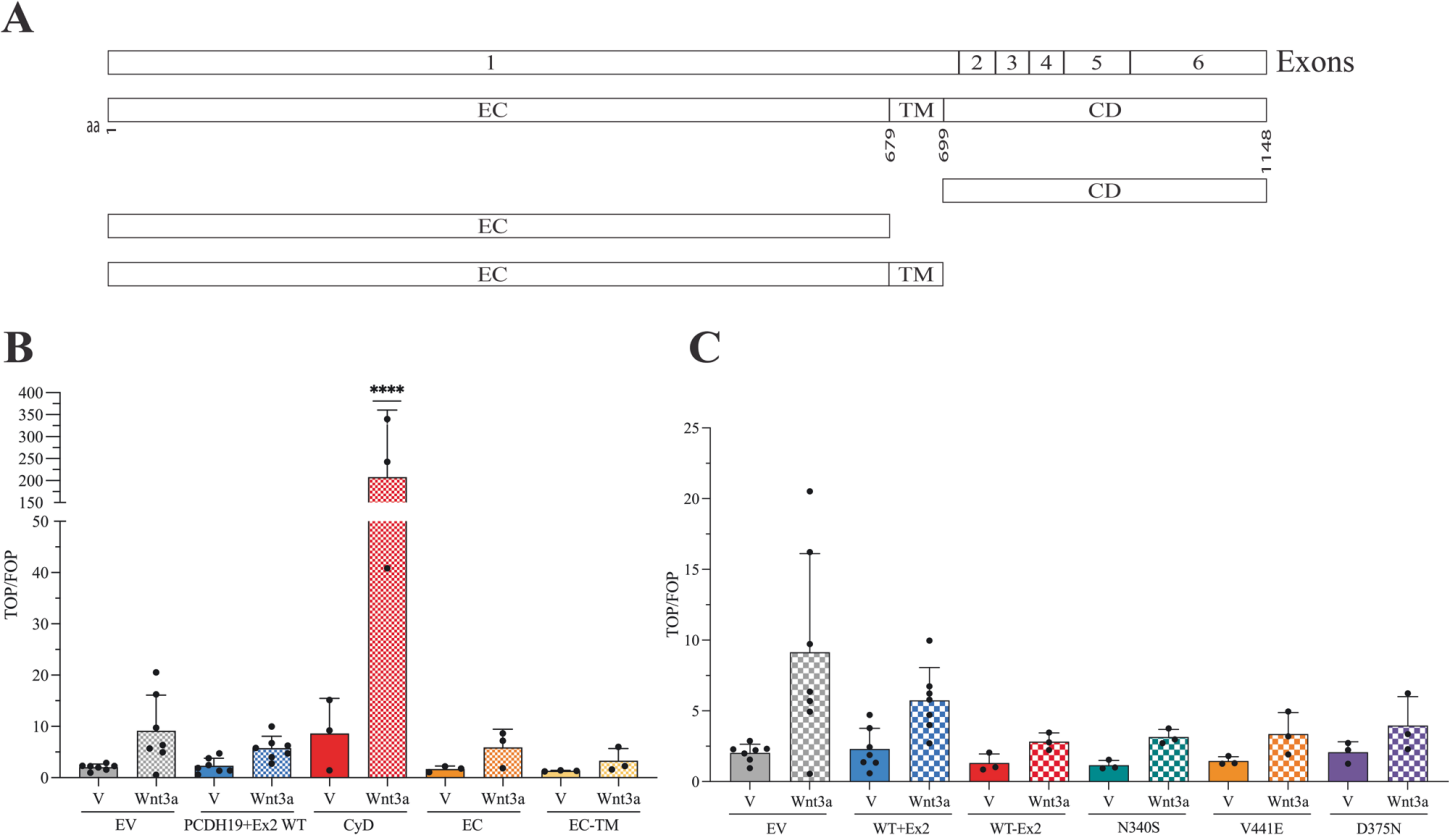
PCDH19 cytoplasmic domain promotes β-catenin transcriptional activity in vitro. **A** Schematic diagram of the PCDH19 WT, cytoplasmic domain (CD), extracellular (EC) and extracellular and transmembrane (EC-TM) fragments used in TOP-Flash reporter assay. **B** HEK293T cells transfected with empty vector (EV), PCDH19 full-length (WT), CD, EC or EC-TM were treated with 100 ng/mL Wnt3a or vehicle (V). Firefly Luciferase values for TOP and FOP reporter activities were normalised to *Renilla* Luciferase transfection control and the TOP/FOP ratio was calculated. Statistical analysis comparing the PCDH19 + Ex2 WT and CD Wnt3a treated samples was performed using One-way ANOVA with Sidak’s correction (****=*p* ≤ 0.0001). **C** HEK293T cells transfected with empty vector (EV), PCDH19 WT (+Ex2 or −Ex2) or CE variant constructs were treated with 100 ng/mL Wnt3a or vehicle (V). Firefly Luciferase values for TOP and FOP reporter activities were normalised to *Renilla* Luciferase transfection control and the TOP/FOP ratio was calculated. The data is presented as mean values ± SD from three technical replicates and at least three biological replicates.

PCDH19 has been found to undergo cleavage by ADAM10 and possibly γ-secretase, with the C-terminal fragment being shown to enter the nucleus and associate with LSD1 to regulate gene expression [6]. We therefore predicted that the cytoplasmic domain of PCDH19 could influence the transcriptional activity of other proteins, such as β-catenin. We performed TOP-Flash reporter assay - that determines the level of Wnt/β-catenin signalling by measuring firefly luciferase activity - in HEK293T cells transfected with constructs expressing the cytoplasmic domain (CD), extracellular cadherins (EC) and extracellular cadherins-transmembrane domain (EC-TM) of human PCDH19 (Fig. 4A) treated with Wnt3a ligand or vehicle to determine the impact of the PCDH19 domains on Wnt/β-catenin signalling. Western blotting confirmed expression of the PCDH19 proteins (Supplementary Fig. 7A). The results showed increased β-catenin transcriptional activity in the cells expressing the PCDH19 CD region, but not full-length, EC or EC-TM proteins (Fig. 4B). TOP-Flash reporter assay of mouse PCDH19 and CD was performed in our collaborator’s laboratory and revealed increased β-catenin transcriptional activity in Wnt3a treated CD- but not PCDH19 full-length- ectopically expressing cells (Supplementary Fig. 8). These results independently confirmed that CD activated β-catenin transcriptional activity, further suggesting a possible role of PCDH19 CD in the regulation of gene expression. We also explored the role of mouse CD in β-catenin-mediated gene regulation through luciferase assay of HEK293T cells transfected with plasmid expressing mutant CD proteins: ΔCM1, ΔCM2 or mutated nuclear localisation signal (NLS). Our results showed a significant decrease in Wnt3a-activated β-catenin transcriptional activity by CD with mutated NLS compared to the CD with wildtype NLS. CM1 and CM2 deletion had no impact on the gene regulatory effect of CD (Supplementary Fig. 8). Overall, the results suggest that the NLS has a vital role in facilitating PCDH19-CD-mediated gene regulation.

To assess the effect of PCDH19 isoforms and CE-variants on β-catenin transcriptional activity, we performed the TOP-flash reporter assay in HEK293T cells transfected with constructs expressing human PCDH19 + Ex2, PCDH19-Ex2 isoforms or CE variant and treated with vehicle or Wnt3a ligand. Expression of the exogenous proteins was confirmed by western blotting (Supplementary Fig. 7B). WT PCDH19 + Ex2 showed no effect on β-catenin transcriptional activity when compared to the empty vector control. However, PCDH19 + Ex2 pathogenic missense variants – p.N340S [2], p.V441E [4] and p.D375N [35] - and PCDH19-Ex2 isoform showed a reduced β-catenin transcriptional activity in the presence of vehicle or Wnt3a that was not statistically significant (Fig. 4C). Therefore, the impact of PCDH19 missense variants and isoform on β-catenin transcriptional activity in this model remains unclear.

**Fig. 5:**
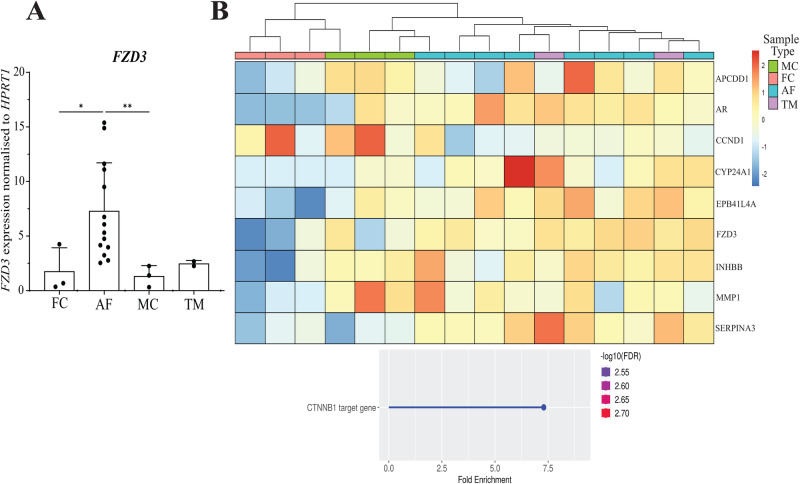
Dysregulation of Wnt/β-catenin signalling in CE patient skin fibroblasts. **A** RT-qPCR analysis of *FZD3* expression in FC (*n* = 3), AF (*n* = 14), MC (*n* = 3) and TM (*n* = 2) skin fibroblasts. Statistical analysis was performed using unpaired t-test with Welch’s correction. *=*p* ≤ 0.05, **=*p* ≤ 0.01. Data presented as mean values ± SD. **B** Hierarchical clustering of MCs, FCs, TMs and AFs expression of Wnt/β-catenin targets and *FZD3* (*above*). These targets are dysregulated in the AF vs FC RNA-sequencing results. Transcription factor target enrichment analysis of AF vs. FC dysregulated genes (*below*).

To determine if the dysregulation of β-catenin targets occurs in CE patient cells, we looked at our existing RNA-sequencing results from RNA isolated from the skin fibroblasts of affected females (AF; *n* = *8*), transmitting males (TM; *n* = *2*) and male (MC; *n* = 3) and female controls (FC; *n* = *3*) [36]. We observed significant upregulation of the Wnt receptor *FZD3* in affected females compared to controls, which we validated by RT-qPCR [37] (Fig. 5A). Gene ontology analysis of differentially expressed genes (DEGs) identified enrichment of genes regulated by β-catenin (*CTNNB1*) when comparing AFs vs. FCs, but not TMs vs. MCs (Fig. 5B, *below*). Hierarchical clustering of the PCDH19 cohort based on significantly dysregulated Wnt/β-catenin targets [*APCDD1* [38]*, AR* [39]*, CCND1* [40]*, CYP24A1* [41], *EPB41L4A* [42], *INHBB* [43]*, MMP1* [44] and *SERPINA3* [45]] and *FZD3* revealed that AFs have Wnt/β-catenin target expression more similar to male than female controls, while TMs cluster among the AFs (Fig. 5B, *above*). Altogether, these results point towards dysregulation of the Wnt/β-catenin signalling pathway in individuals with pathogenic PCDH19 variants.

## Discussion

Pathogenic *PCDH19* variants cause the debilitating neurological disorder CE [4]. This brain-specific phenotype of CE is likely caused by PCDH19’s high expression in the brain and low expression in peripheral tissues [46]. It is therefore important to study the PCDH19 proteome in the mammalian brain. Here we present PCDH19 interactome data measured using LC-MS/MS technique from the developing mouse brain from two independent experiments. None of the potential interacting proteins identified in Experiment 1 overlapped with those identified in Experiment 2. This could be due to differences in the controls used (untagged mouse tissues vs. mouse IgG beads) and the small number of proteins identified, perhaps due to the limited amount of IPed samples. However, our experimental results confirmed the interaction between PCDH19-β-catenin (Experiment 1) and PCDH19-CAPZA1 and PCDH19-αN-catenin (Experiment 2), suggesting that both the methods lead to identification of proteins that could be validated individually. Our experimental and meta-analysis results showed an enrichment of proteins involved in cytoskeleton organisation, actin binding and neurogenesis (Fig. 2) that are consistent with the known functions of PCDH19 in cell adhesion and mobility [11, 12, 17], as well as pointing to novel pathways that are potentially involved in CE disease mechanism [47].

Overall, our proteomic and gene regulatory studies suggest the role of PCDH19 in the Wnt/β-catenin signalling pathway. This pathway has been reported to be dysregulated in a range of disorders such as autism spectrum disorder (ASD), schizophrenia and epilepsy, and is involved in regulating neurogenesis [48]. Furthermore, various studies have reported that β-catenin and Wnt ligands are upregulated post-seizure in rats, and mice that lack *Ctnnb1* in the hippocampus and cortex and have increased susceptibility to pentylenetetraxole (PTZ) induced-seizures [48]. Many recent studies in *Pcdh19*^*+/KO*^ mice and *pcdh19*^*−/−*^ zebrafish have linked the canonical (β-catenin dependent) and non-canonical (β-catenin independent) Wnt pathways to CE pathogenesis [11, 15, 34]. Our transcriptomic and proteomic studies have identified the involvement of this pathway in CE from a systemic perspective. Aiming at gathering additional experimental evidence supporting the role of β-catenin in CE, we have shown that PCDH19 interacts with β-catenin in both the mammalian brain and human cells. The strength of this interaction is not significantly affected by the PCDH19 isoform or CE variants. However, our transcriptomic assays showed that PCDH19 CE variant may affect the transcriptional activity of β-catenin. Unsurprisingly, our TOP-Flash results showed that the PCDH19 cytoplasmic domain increases β-catenin transcriptional activity. As this domain has also previously been shown to regulate LSD1 transcriptional activity and nuclear localisation [6, 7], accumulating evidence is pointing to cleaved PCDH19 CD as an important transcription co-regulator. Overall, our data has linked PCDH19 with the transcriptional role of β-catenin.

**Fig. 6:**
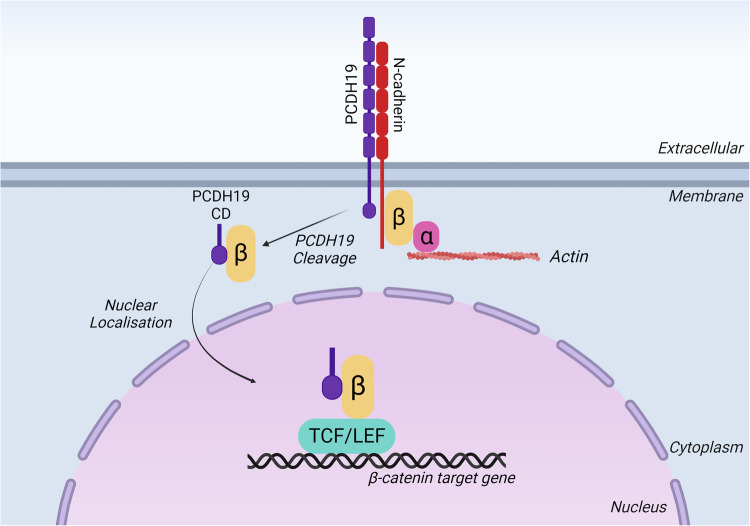
Proposed model summarising the interplay between PCDH19 and β-catenin. PCDH19 protein exists in the cell membrane in a complex with N-cadherin and β-catenin (β) to mediate cell-cell adhesion. αN-catenin (α) links this complex to the actin cytoskeleton. The cytoplasmic domain (CD) of PCDH19 is cleaved by ADAM10 [6]. PCDH19 CD and β-catenin localise to the nucleus to activate gene expression via the transcription factors TCF/LEF.

Our results suggest that PCDH19 interacts with multiple actin binding and regulatory proteins. One potential mechanism linking PCDH19 to the actin cytoskeleton is through the N-cadherin, α-catenin, and β-catenin complex. Ca^2+^-dependent cell adhesion is facilitated by the interaction between β-catenin and cadherins such as N-cadherin. This interaction is mediated by the binding of α-catenin to β-catenin which results in the formation of the α-catenin/β-catenin heterodimer [30]. This allows β-catenin to bind to N-cadherin, thus promoting cadherin-based cell adhesion. α-catenin is also able to promote actin bundling through its force-dependent interaction with filamentous actin (F-actin) [30]. The functional outcome of α-catenin binding depends on its dimerisation. Monomeric α-catenin binds strongly to β-catenin while homodimeric α-catenin preferentially binds actin filaments [30]. Therefore, the binding of α-catenin to β-catenin negatively regulates actin polymerisation by preventing α-catenin homodimerization and actin bundling [30]. As PCDH19 interacts with α-catenin, β-catenin and N-cadherin [10], we propose that PCDH19 is an important part of this complex, contributing to the formation of stable adhesion junctions (Fig. 6).

Our results indicate that PCDH19 CE-variants can influence the Wnt/β-catenin signalling pathway. Although not reaching statistical significance, our TOP-Flash reporter results showed that Wnt signalling is reduced on PCDH19 CE variant overexpression. We note, however, that the TOP-Flash reporter assay only measures the transcriptional activity of β-catenin complexed to TCF/LEF transcription factors caused by Wnt signalling, although β-catenin can bind to many transcription factors (for example FOXOs, nuclear receptors, SOX and SMAD) [49]. On the other hand, our RNA-seq results from the skin fibroblasts of CE individuals (and results of Biswas *et al*. 2021 in *pcdh19*^*−/−*^ zebrafish) [15] indicate upregulation of some Wnt/β-catenin targets. Whether these observations can be due to cell type or developmental stage-specific effects of Wnt/β-catenin signalling needs to be investigated [49, 50]. Nevertheless, this pathway can be modulated via a range of compounds, for example the anti-seizure medication valproic acid [48] (sometimes prescribed to CE patients [51]) and lithium chloride [used for treating mood disorders] [48, 52], thus making Wnt/β-catenin signalling a potential target for the development of novel anti-seizure medication. Therefore, further studies investigating the Wnt/β-catenin pathway in CE individuals are required.

Overall, we conclude that our proteomic analysis of the affinity-purified PCDH19 complexes from the mouse brain has identified CAPZA1, αN-catenin and β-catenin as PCDH19 interacting proteins that play critical roles in brain development, neuron migration and differentiation. Functional association of many other PCDH19 interacting proteins that we have identified here and that have roles in disease-relevant pathways will need further investigation. Our results shed light on the expanding role of PCDH19 in association with multiple proteins and complex pathways.

## Supplementary information

Supplementary Information

Supplementary Table 1

Supplementary Table 2

Supplementary Table 3

Supplementary Table 4

## Data Availability

All codes are available upon request.
