# Supplementary material for: Proteomic analysis of the developing mammalian brain links PCDH19 to the Wnt/β-catenin signalling pathway
Source: Mol Psychiatry. 2024 Mar 7;29(7):2199–210. doi: 10.1038/s41380-024-02482-z (PMC11408250; doi:10.1038/s41380-024-02482-z)
Supplement: Supplementary file 1 — Supplementary Information [file 41380_2024_2482_MOESM1_ESM.docx]

**SUPPLEMENTARY INFORMATION**

**Proteomic analysis of the developing mammalian brain links PCDH19 to the Wnt/β-catenin signalling pathway.**

Rebekah de Nys^1,2^, Alison Gardner^1,2^, Clare van Eyk^1,2^, Stefka Mincheva-Tasheva^1,2,3,4^, Paul Thomas^1,2,3,4^, Rudrarup Bhattacharjee^1,2^, Lachlan Jolly^1,2^, Isabel Martinez-Garay^5^, Ian W. J. Fox^5^, Karthik Shantharam Kamath^6^, Raman Kumar^1,2, Ϯ^ and Jozef Gecz^1,2,4,7, Ϯ,*^

^1^ Adelaide Medical School, The University of Adelaide, Adelaide, SA, Australia.

^2^ Robinson Research Institute, The University of Adelaide, Adelaide, SA, Australia.

^3^ Genome Editing Program, Adelaide, SA, Australia

^4^ South Australian Health and Medical Research Institute, Adelaide, SA, Australia

^5^ Division of Neuroscience, School of Biosciences, Cardiff University, Cardiff, Wales, United Kingdom

^6^ Australian Proteome Analysis Facility, Macquarie University, Macquarie Park, NSW, Australia

^7^ School of Biological Sciences, The University of Adelaide, Adelaide, SA, Australia

^Ϯ^ Co-last authors

*Corresponding author: Email: [jozef.gecz@adelaide.edu.au](mailto:jozef.gecz@adelaide.edu.au), Phone: 61 8 8313 2453, Address: Adelaide Health and Medical Sciences Building, Level 8, 4 North Terrace, Adelaide, SA, Australia.

**SUPPLEMENTARY FIGURES**

**Supplementary Fig. 1 Schematic diagram of the mRNA and protein domains of PCDH19+Ex2.** PCDH19 contains a signal peptide (SP), extracellular cadherin repeats (EC1-6), transmembrane (TM) and conserved CM1 and CM2 domains. Nuclear localisation signal (NLS) 760-782aa was experimentally validated while NLSs 1031-1038aa and 1138-1145aa are predicted ^6, 7^. The binding regions (where known) of experimentally validated PCDH19 interacting proteins are also shown. PCDH19 interactions with N-cadherin, PCDH19, NONO, DOCK7, NEDD1, PCDH17 and PCDH10 were identified in human cell lines (purple) ^7, 10-12^. PCDH19 interactions with LSD1 and GABA_A_R alpha 1 were identified in both rat brain and human cell lines (green-purple) ^6, 9^. PCDH19 interactions with NAP1 and CYFIP2 were identified in chicken brain (yellow) ^13^. PCDH19 interactions with β-catenin, CAPZA1 and αN-catenin were identified in mouse brain and human cell lines (blue-purple) (*this study*).

**
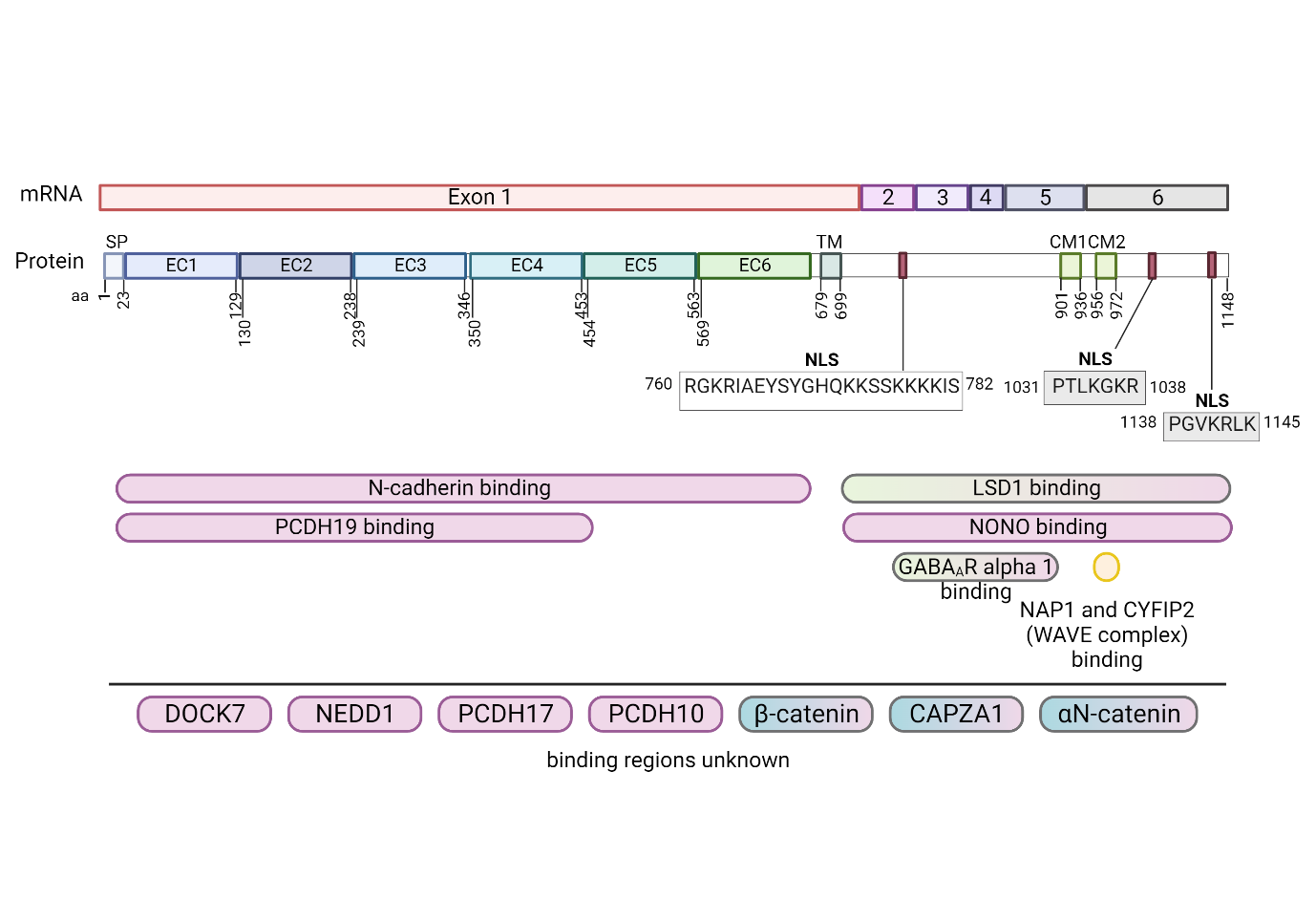
**

**Supplementary Fig. 2** PCDH19 interaction with N-cadherin. Lysate from the hippocampi/cortices of **(A)** embryonic (E17.5-18.5) or **(B)** postnatal (P6-7) PCDH19-HA-FLAG mice was incubated with mouse IgG or anti-FLAG agarose beads. Input (2%) and IP (20%) samples were western blotted to detect PCDH19 and N-cadherin. Mouse IgG beads showed a low level of non-specific N-cadherin signal.


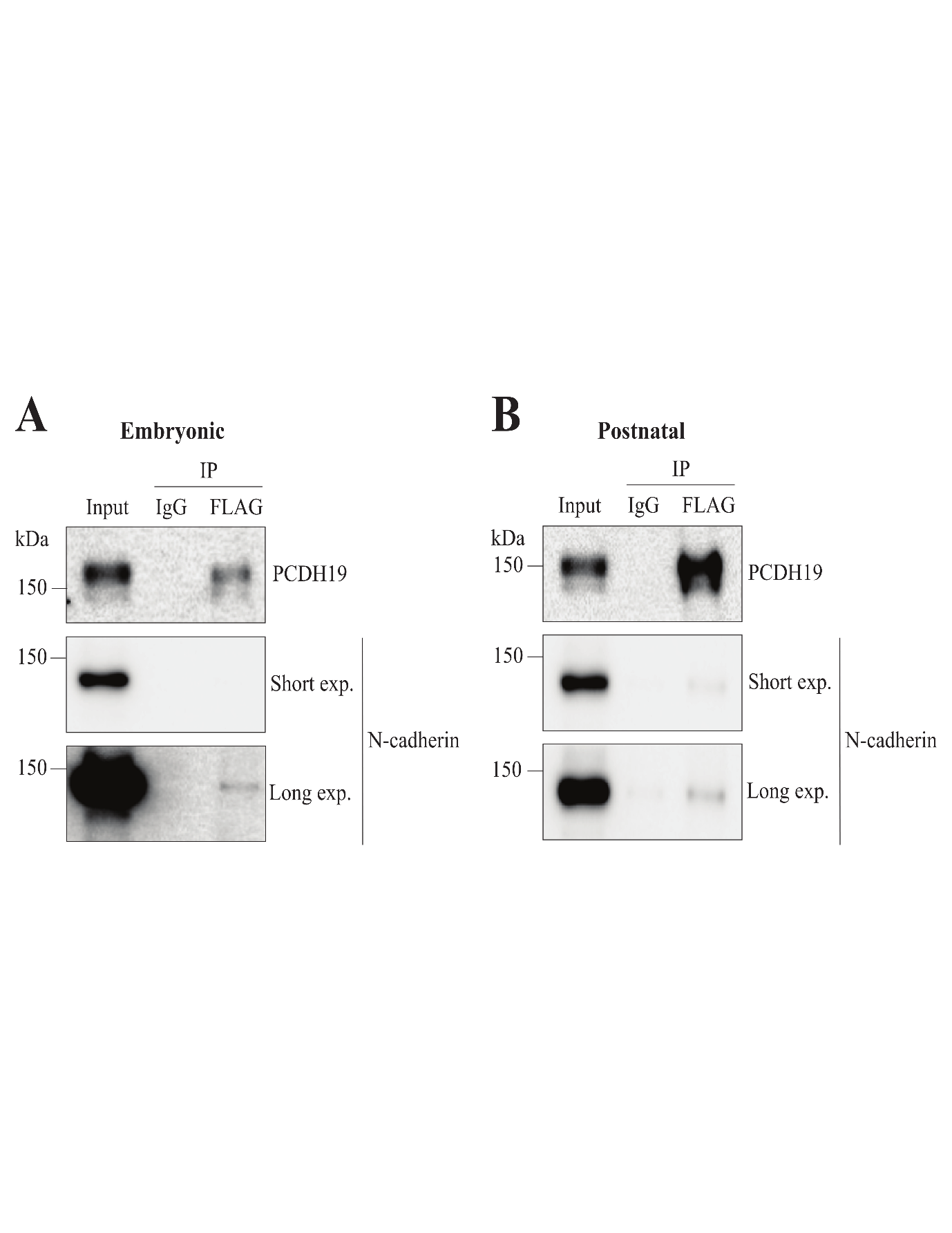


**Supplementary Fig. 3 Enrichment analysis of the known PCDH19 interactome.** **A** Bar graph of the models used for PCDH19 proteome analysis. **B** Molecular pathway, **C** Cellular Component, **D** Biological Process, **E** Subcellular Component ^17^, and **F** Signalling pathway enrichment analysis ^19^ of all currently identified interacting proteins. The graphs show the top 10 enriched pathways sorted by FDR and Fold enrichment (B-E) or Combined Score (F)*.* n= number of genes, FE= fold enrichment, CS= combined score.

**
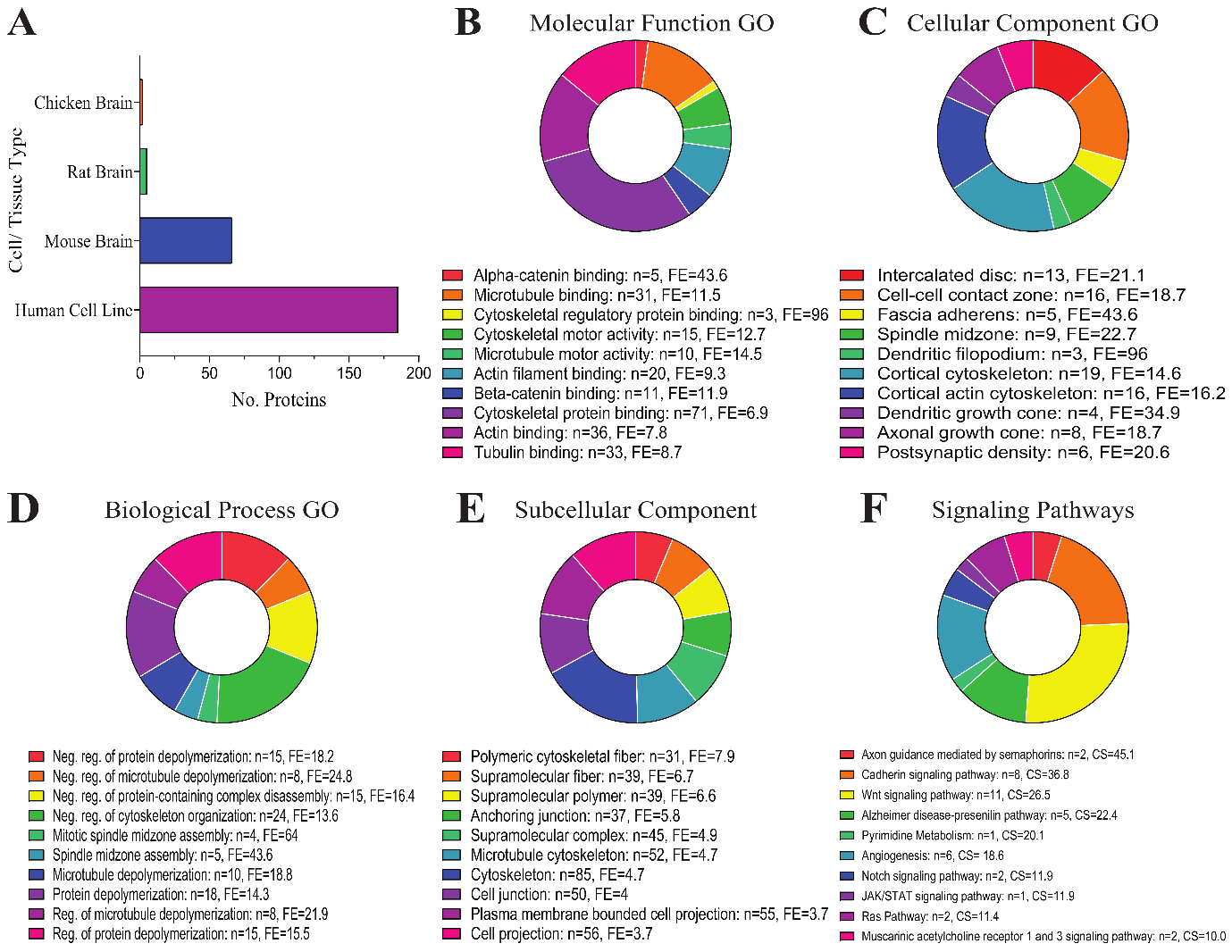
**

**Supplementary Fig. 4 CAPZA1, αN-catenin and β-catenin interaction with δ2-PCDHs.** CAPZA1-HA was immunoprecipitated with anti-HA agarose beads. Inputs (2%) and IP (25%) samples were western blotted to detect **A-C** CAPZA1-HA and **A** PCDH10-Myc, **B** PCDH12-Myc and **C** PCDH17-Myc. **D** PCDH10-Myc, **E** PCDH12-Myc and **F** PCDH17-Myc were immunoprecipitated with anti-Myc magnetic beads. Inputs and IP samples were western blotted to detect **D-F** Myc-tagged proteins and αN-catenin-HA. **G** PCDH10-HA, **H** PCDH12-HA were immunoprecipitated with anti-HA agarose beads and **I** PCDH17-Myc was immunoprecipitated with anti-Myc magnetic beads. Inputs and IP samples were western blotted to detect **G-I** Myc-tagged, HA-tagged and β-catenin-V5 proteins.

**
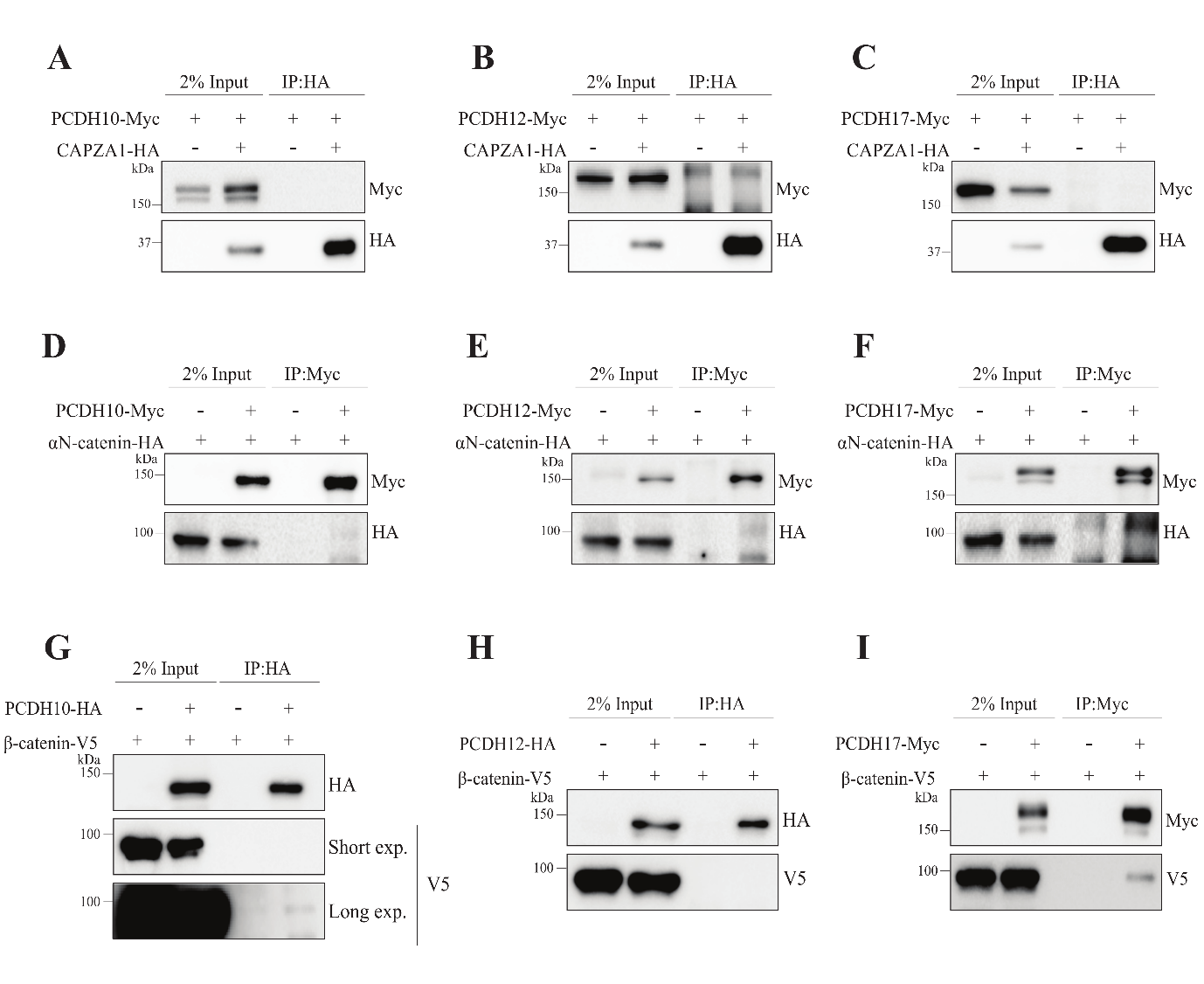
**

**Supplementary Fig. 5** Myc-PCDH19+Ex2-FLAG, Myc-PDH19 CD-FLAG, Myc-PCDH19 EC-FLAG and Myc-PCDH19 EC-TM-FLAG domains were immunoprecipitated with anti-FLAG beads. Inputs (2%) and IP (25%) samples were western blotted to detect exogenous Myc-PCDH19-FLAG and β-catenin-V5 proteins.

**
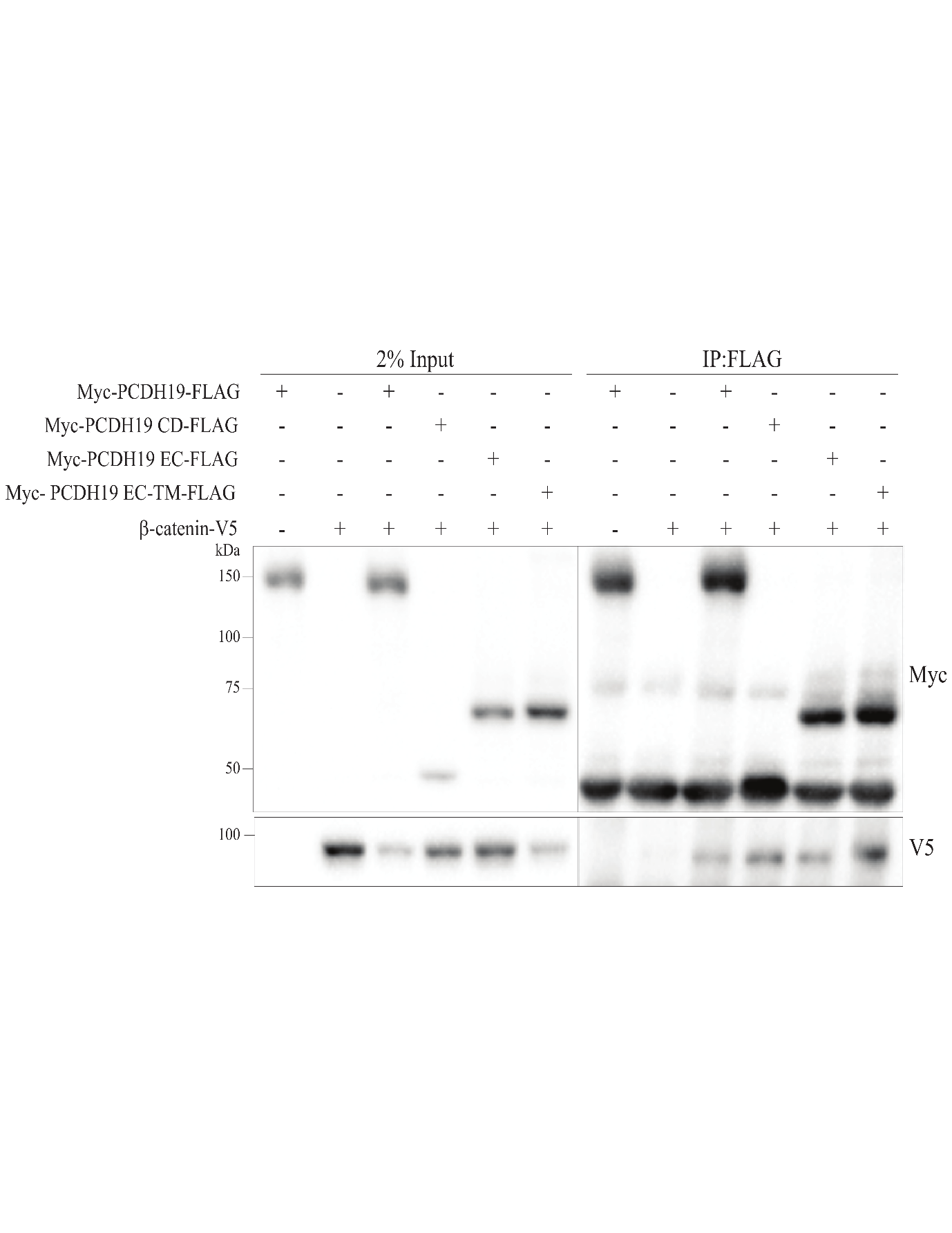
**

**Supplementary Fig. 6** Myc-PCDH19 Full Length (FL)-FLAG and Myc-PCDH19 EC (Extracellular Domain)-FLAG were immunoprecipitated with anti-FLAG conjugated agarose beads from HEK293T cells expression one or more tagged protein as shown. Inputs and IP samples were western blotted to detect Myc-PCDH19 FL-FLAG, Myc-PCDH19 EC-FLAG, N-cadherin (CDH2)-HA and β-catenin-V5.





**Supplementary Fig. 7** Western blot of HEK293T cells transfected with **(A)** empty vector (EV), PCDH19+Ex2 WT, CD, EC or EC-TM proteins, or **(B)** empty vector (EV), PCDH19+Ex2, PCDH19-Ex2 or CE missense variant proteins were probed with anti-Myc and anti-β-Tubulin (loading control) antibodies.


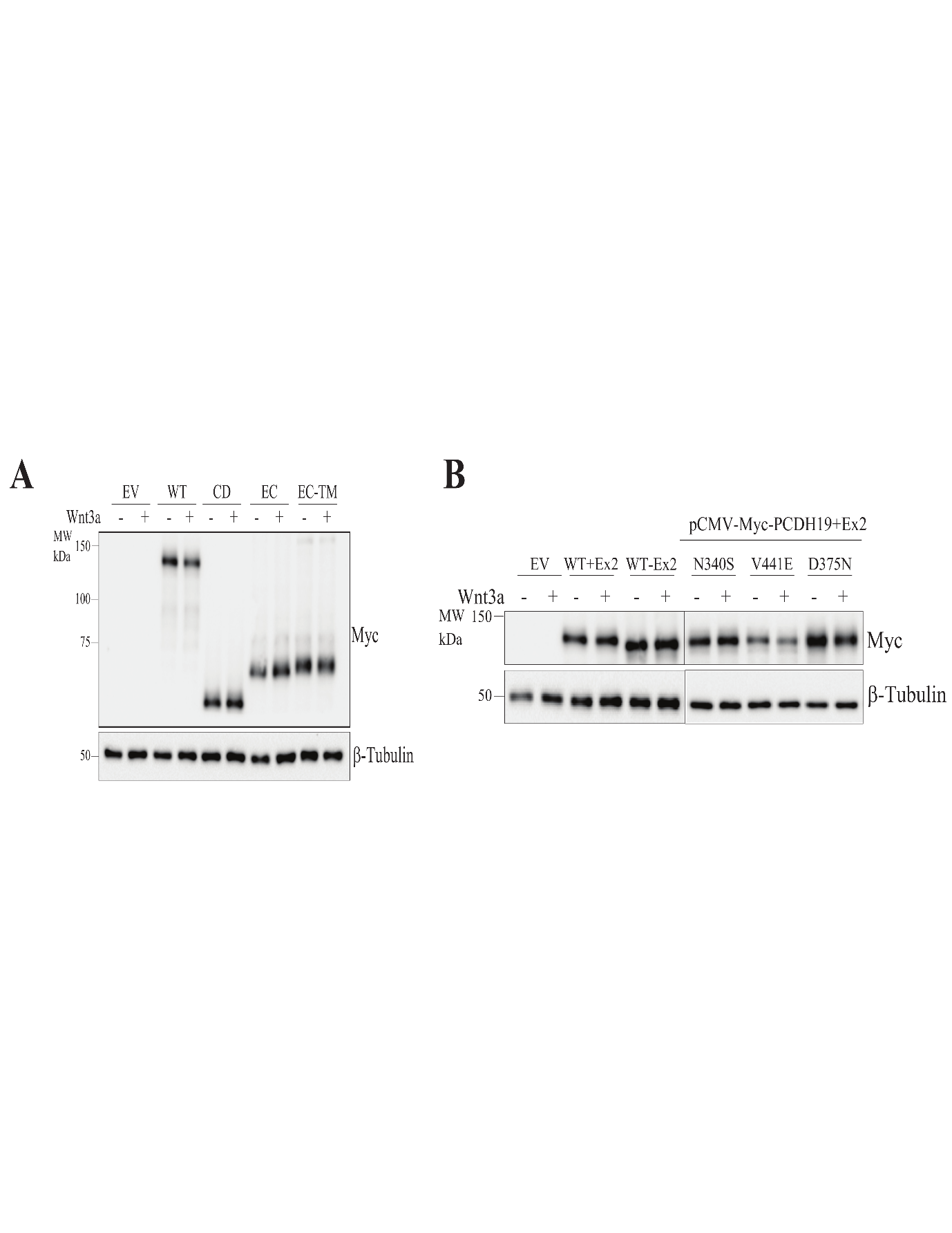


**Supplementary Fig. 8 A** Schematic diagram of the mouse full length PCDH19+Ex2 WT, cytoplasmic domain (CD), cytoplasmic domain deleted CMI (ΔCM1), CM2 (ΔCM2) or mutated nuclear localisation signal (MT-NLS) used in TOP-Flash reporter assay. **B** HEK293T cells were transfected with empty vector (EV) or mouse full-length PCDH19 (WT+Ex2), CD, ΔCM1, ΔCM2 or MT-NLS expression constructs and treated with 200 ng/mL Wnt3a or vehicle (V) for 24 hours. Firefly Luciferase values for TOP were normalised to *Renilla* Luciferase transfection control. Statistical analysis was performed using Two-way ANOVA with Sidak’s correction (****=p ≤0.0001). Data presented as mean values ± SD Each experiment was performed in two technical replicates and seven biological replicates. **C** Western blot of HEK293T cells transfected with empty vector (EV), mouse PCDH19+Ex2 (WT+Ex2), CD, ΔCM1, ΔCM2 or MT-NLS expression constructs were probed with anti-HA and anti-GAPDH (loading control) antibodies.


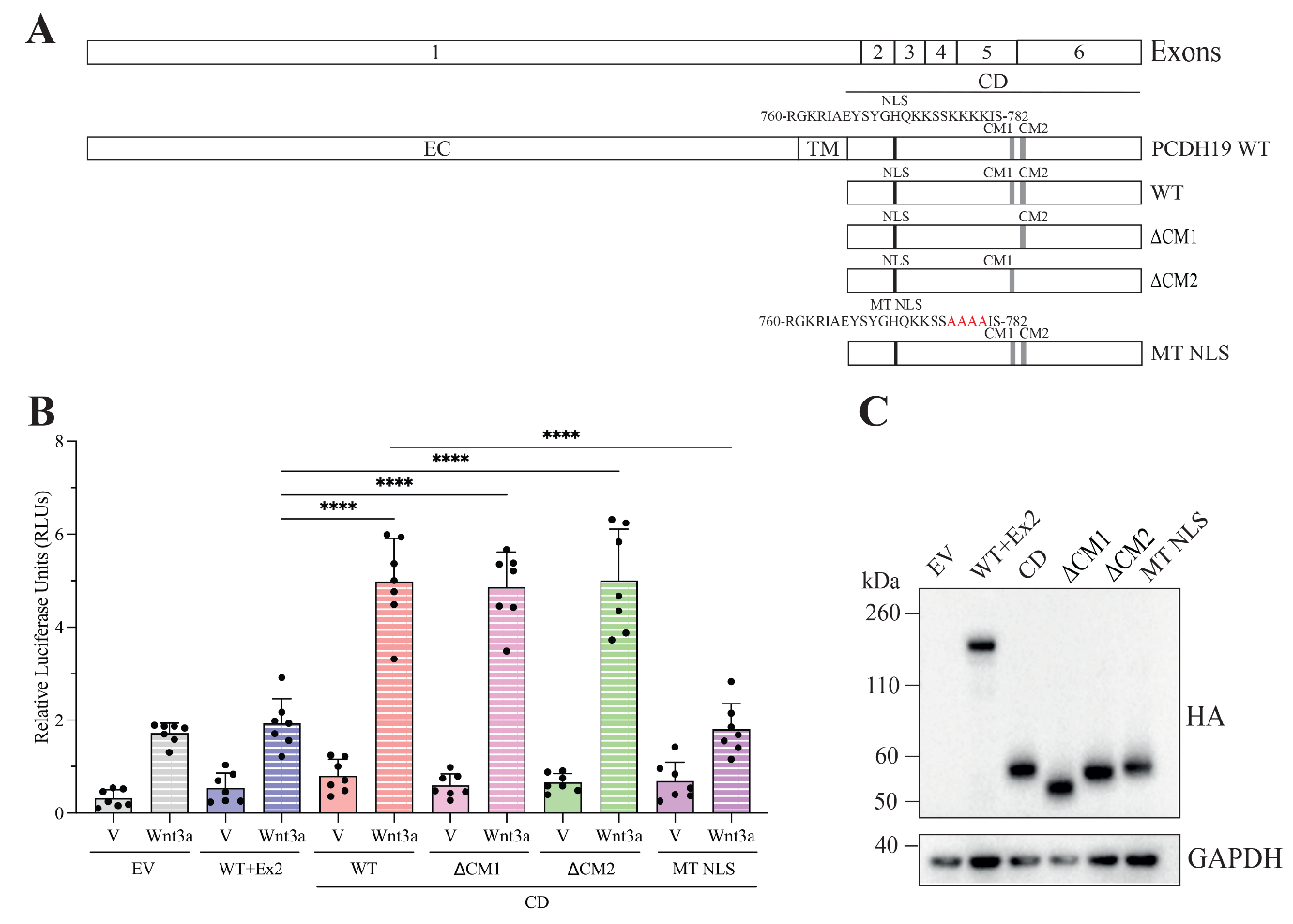


**SUPPLEMENTARY TABLES (Provided as separate files)**

**Supplementary Table 1** Interactome data from embryonic PCDH19-HA-FLAG and untagged mouse cortices.

**Supplementary Table 2** Interactome data from embryonic and postnatal PCDH19-HA-FLAG mouse hippocampi and cortices.

**Supplementary Table 3** Disease-associated potential PCDH19 interacting proteins. List of PCDH19 interacting proteins identified in the mouse brain associated with OMIM diseases.

**Supplementary Table 4** Meta-analysis of reported PCDH19 interacting proteins.

**SUPPLEMENTARY METHODS**

**Supplementary Methods Table:** List of primers used for cloning CTNNA2, CAPZA1 and CDH2 into pCMV-HA vector.

| **Primer Name** | **Sequence (5’-3’)** |
| --- | --- |
| hCTNNA2-InFus_F | GCGATCGCGATATCAAGCTTATGACTTCGGCAACTTCACCTATC |
| hCTNNA2-InFus_R | ATGTCTGGATCCCCGCGGCCGCCTAGAAGGAATCCATTGCTTTGAATTC |
| hCAPZA1-InFus_F | GCGATCGCGATATCAAGCTTATGGCCGACTTCGATGATCGTGTG |
| hCAPZA1-InFus_R | ATGTCTGGATCCCCGCGGCCGCTTAAGCATTCTGCATTTCTTTGC |
| hCDH2_InFus_F | AGGCCCGAATTCGCGATCGCGATATCATGTGCCGGATAGCGGGAGCGCTGCGGA |
| hCDH2_InFus_R | CCGCGGCCGCGGTACCTCGAGAGTCAGTCATCACCTCCACCATACATGTCA |
